# Multidimensional Scaling Analysis of the Dynamics of a Country Economy

**DOI:** 10.1155/2013/594587

**Published:** 2013-10-31

**Authors:** J. A. Tenreiro Machado, Maria Eugénia Mata

**Affiliations:** ^1^Department of Electrical Engineering, Institute of Engineering, Polytechnic of Porto, Rua Dr. António Bernardino de Almeida 431, 4200-072 Porto, Portugal; ^2^Nova SBE, Faculdade de Economia, Universidade Nova de Lisboa, Campus de Campolide, Travessa Estevão Pinto, 1099-032 Lisbon, Portugal

## Abstract

This paper analyzes the Portuguese short-run business cycles over the last 150 years and presents the multidimensional scaling (MDS) for visualizing the results. The analytical and numerical assessment of this long-run perspective reveals periods with close connections between the macroeconomic variables related to government accounts equilibrium, balance of payments equilibrium, and economic growth. The MDS method is adopted for a quantitative statistical analysis. In this way, similarity clusters of several historical periods emerge in the MDS maps, namely, in identifying similarities and dissimilarities that identify periods of prosperity and crises, growth, and stagnation. Such features are major aspects of collective national achievement, to which can be associated the impact of international problems such as the World Wars, the Great Depression, or the current global financial crisis, as well as national events in the context of broad political blueprints for the Portuguese society in the rising globalization process.

## 1. Introduction

Debt crises and defaults have been the subject of extensive studies during the recent decades. Capie and Wood [1] offer the distinction between banking and currency crises. More recently, Kindleberger [2] lists all crises until 1998, while Eichengreen et al. [3] address the Latin America and southern periphery of Europe. Bordo [4] develops a mismatch analysis for public deficit and balance of payments, while Reinhart and Rogoff [5] pay attention to the current global financial crisis, after dealing with banking, financial, and debt crises [6–8]. Portuguese economic historians have long studied the state of affairs of the contemporary national economy as Tengarrinha's [9] guide list for readers clearly shows. Portugal remains obscure in the prevailing historiography regarding the overview for crises throughout the nineteenth and twentieth centuries. In fact, crises are key issues for understanding the main structure of the Portuguese economy and, to some extent, of the European economy. Moreover, the current Portuguese crisis case study may foster greater global curiosity about the long-run anatomy of peripheral partners' economic bottlenecks. Is a Portuguese bankruptcy now unavoidable? What consequences will come out of the current situation?

**Figure 1:**
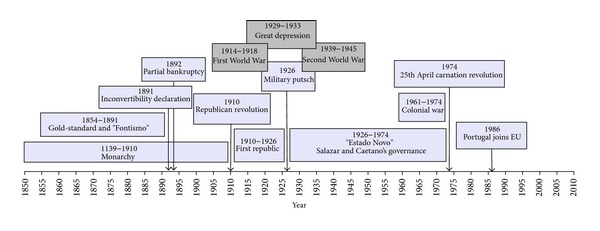
Timeline of Portuguese history over the last 150 years.

Focusing on only one country brings advantages related to greater expertise and more precise knowledge on the quality of sources and reliability of data [10]. Moreover, Portugal is today a case of interest in the current global crisis context. Smallness, difficulties in foreign trade competition, and weakness of productive sectors are the main and most explored reasons for the late-nineteenth-century industrialization.  Figure 1 is a timeline of Portuguese history over the last century and a half.

Many researchers dwell on the abandonment of the gold-standard in 1891 and chronic, severe financial problems as major topics of Portuguese historiography [11–15]. While the first quarter of the twentieth century is of historical interest regarding politics and the financial effects of Republican governance [16, 17], the success of the modern economic growth from the Great Depression to the 1970s, under the “Estado Novo” political regime, also has drawn considerable attention [18–20]. Given the structural economic conditions following the 1846-47 civil war, long-run studies on short-run crises in the decades of 1860, 1870, and 1880 could be expected to lead to the decision to abandon the gold-standard [21], but twentieth-century crises never received a long-run analysis. Moreover, the approach for the whole set of Portuguese crises throughout the contemporary age has never been attempted. This paper is devoted to the Portuguese business cycles. As the available literature uses empirical approaches, a formal mathematical analysis is used here. In using the MDS methodology, the economy is considered as a system, and the elected variables are used as signals. In a second step, the mathematical results that are obtained are checked in comparing similarities and dissimilarities with qualitative historical evidence.

The difficulties of such an exercise have to do with significant breaks of societal structure concerning the impact of global events, namely, the two World Wars and the Great Depression, and the meaning of national projects, such as the building of a new colonial empire in Africa, following the 1880s' Berlin Conference (which was decolonized in 1975), or the succession of several government political blueprints. Such daunting difficulties have discouraged the possibility of embracing the analytical purpose of such a total overview for Portuguese crises, preventing the study of this national 2007–2011 crisis from the centennial historical perspective.

The purpose of this paper is to present such an attempt, resorting to a statistical reconstruction of the evolution of the Portuguese economy from the mid-1860s to the Second World War and the official data from then until now. In fact, the interwar period saw significant improvements in Portugal's official statistics, leading to, among other things, the publication of official data about the Portuguese balance of payments after the Second World War. This means that the work of reconstruction became almost pointless from 1948 onwards. The methodology departs from setting a vector of the available macroeconomic variables for each year, which are common to all periods of time, and utilizes the Multidimensional Scaling (MDS) statistical methodology, in order to obtain a measure of similarity or “likeness” for years and periods of time, letting the data speak for itself. This method was already applied to identify US crises [22] and stock-market index fluctuations [23, 24]. It uses measures for similarity (or, alternatively, of dissimilarity) between the vectors, comprising values for gross domestic product (GDP), exports, imports, fiscal revenue, and effective Public Expenditure. These variables cover not only the public deficit and public debt, but also capture international trade, productive specialization, and economic growth. GDP per capita is gross domestic product divided by population. The trade deficit is estimated from imports and exports. Public deficit is estimated from government revenues and expenditures. Before 1998, all the variables were collected from The Portuguese Historical Statistics [25]. For the last years, they were collected from “Anuários Estatísticos,” “INE,” and “Conta Gerais do Estado” [26, 27]. One might consider Laíns foreign trade data [28]. However, Laíns data stop at 1914. Moreover, they do not match other items of the Portuguese balance of payments. GDP per capita for Portugal from [29] reaches similar conclusions on growth and economic performance. Large public deficits are associated with larger public goods provision, crowding-out effects, and public debt accumulation. Large trade deficits are associated with foreign dependence for domestic consumption, and also with dependence on foreign decisions that drive other items of the balance of payments, such as other countries' foreign direct investment decisions and emigrants' remittance flows. The variables selected address governmental financial activity and foreign trade and are considered both in the Neoclassical and Keynesian paradigms as key factors for GDP performance.

Public and foreign imbalances may have occurred alone or alltogether, depending on the character of the years and periods. When occurring together, these *twin crises* suffer from more difficult economic-policy correction mechanisms (as [4] demonstrates). Two vector normalizations were introduced in order to consider the population variation and the absolute interval of each of the adopted variables. The results obtained are quite interesting and provide a very plausible picture for the character and similarity of the Portuguese crises.

This may be a fair provisional description of a few important aspects of Portugal's social and economic life of the late nineteenth and twentieth centuries that also embraces the earliest decade of the new millennium. Of course, plausibility and coherence for the whole picture are not proofs that interpretations here explain the crises described. However, other plausible outcomes that accommodate the chronology, duration, frequency, and character of the crises are not easy to propose. Note that Bordo et al. [30] conclude that in a 21-country sample, there occurred twice as many crises after 1973 as in the fixed-exchange-rate regimes of gold-standard and Bretton Woods, while the 1920s–30s were the most unstable years regarding crises.

The choice of the period under study is justified by statistical and economic reasons. The mid-1860s saw the beginning of a new era in Portugal's statistical life. The first population census occurred in 1864 and the foreign trade statistics began their regular publication in 1865. This means that for earlier periods the basic data for the statistical analysis are almost completely lacking. At the same time, it seems that this was the moment of the first attempt at a take-off of modern economic growth in Portugal, although it failed in the long run [31]. Computations herein are based on the official data and on the available historical estimations for the variables before the Second World War, under plausible hypotheses [32]. The 150-year perspective presented here may help to understand if businessn cycles became more frequent throughout Portugal's modern economic growth process.

Bearing these thoughts in mind, Section 2 formulates the mathematical method in detail; Sections 3 and 4 present the results, and an historical interpretations, respectively. Finally, Section 5 draws the main conclusions.

## 2. Multidimensional Scaling

MDS produces a spatial representation by considering similarity between objects through relatedness [33–35]. For constructing the maps, MDS requires input data in the form of a matrix of similarities between objects based on some comparison criteria to be defined by the user. There are several packages that implement the MDS algorithm and we can mention MATLAB [36, 37], *R* [38, 39], and GGobi [40]. While MATLAB is proprietary, *R* and GGobi are open source. Users can visualize the “maps” produced by MDS and trace conclusions by analysing the relative positions of points and clusters emerging in the representation. In this paper, a vector of values for economic variables is considered [41]. Therefore, objects stand for the economical variables during a given time period, and similarity identifies the degree of “likeness” between two objects [42].

Let us suppose, just for starting, that we have correlation and distance in the common sense. When the correlation is one, the distance between the two points under consideration is zero, and one cannot distinguish them on the MDS map. If two points (in our case, corresponding to the economic variables in two distinct time periods) are located close via the MDS procedure, it means that there is a high correlation between the vectors that produced them. Their similarity means that, if one point represents a severe crisis, the other may be considered as well [43].

MDS requires input data in the form of a matrix of item-to-item correlation matrix *R* and runs an iterative numerical algorithm for estimating the coordinates of the original objects (i.e., economic variables at specific sampling periods) in a given space [44]. Additionally, we can plot and visualize the output MDS map. The lengths adopted for the time periods are *h*, capturing crises associated with the so-called short-run business-cycle fluctuations. Time lags for reacting to market situations may occur, due to information costs in market situations of shortage to face an increase of demand that require the allocation of more productive resources to increase domestic supply in order to match the balance of payments equilibrium and currency exchange-rate stability. Time lags may also occur in situations close to full-employment of productive resources and flood of commodities in the market, as accumulated stocks and inventories require the appropriate opposed decisions, which decrease fiscal revenues, bring government-budget disequilibrium, and increase accumulated public debt [45]. As producers' decisions also take time to materialize, mismatches may occur, leading to significant production and employment fluctuations [46].

As each period is represented as a point in a high-dimensional space, the closer the points, the greater the similarity between them. In other words, as mentioned, points corresponding to similar objects are located quite together, while points corresponding to dissimilar objects are located far apart. Herein after we adopt two measuring indices, namely, the Cosine correlation *r* ^*C*^ and the Euclidean distance *r* ^*E*^:

(1) $$\begin{array}{c} r_{ij}^{C}=\frac{\sum_{t=1}^{h}\sum_{k=1}^{k_{\max}}x_{i}\left(k,t\right)x_{j}\left(k,t\right)}{\sqrt{\sum_{t=1}^{h}\sum_{k=1}^{k_{\max}}x_{i}^{2}\left(k,t\right)\cdot \sum_{t=1}^{h}\sum_{k=1}^{k_{\max}}x_{j}^{2}\left(k,t\right)}},i,j=1,\ldots ,p, \end{array}$$

(2) $$\begin{array}{c} \delta_{ij}^{E}=\sqrt{\overset{h}{\underset{t=1}{\sum }}\overset{k_{\max}}{\underset{k=1}{\sum }}{\left[x_{i}\left(k,t\right)-x_{j}\left(k,t\right)\right]}^{2}},\;i,j=1,\ldots ,p,r_{ij}^{E}=1-\frac{\delta_{ij}^{E}}{\max_{i,j}\left(\delta_{ij}^{E}\right)},\;i,j=1,\ldots ,p, \end{array}$$

where *x*_*i*_ and *x*_*j*_ are the *i*th and *j*th signals of dimension *k*_max⁡_, *t* represents time, *T* is the maximum period of time, *h* the sampling period, and *p* denotes the total number of signals under comparison (in our case *p* = *T*/*h*). The signals *x*_*i*_(*k*, *t*) correspond to the initial time series with a normalization based on the population size *P*(*t*), that is, by performing the ratio *x*_*i*_(*k*, *t*) ← *x*_*i*_(*k*, *t*)/*P*(*t*). The operator max⁡(*δ*_*ij*_^*E*^) gives the maximum value of *δ*_*ij*_^*E*^, so that we get 0 ≤ *r*_*ij*_^*E*^ ≤ 1 in matrix *R*.

For expression (1), we adopt a second normalization step prior to the calculation of *r* ^*C*^ that consists of converting all vector components to the interval between zero and one, that is, by performing for each component *k* the ratio *x*_*i*_(*k*, *t*) ← *x*_*i*_(*k*, *t*)/max⁡_*t*_⁡[*x*_*i*_(*k*, *t*)] between the component value and its maximum value along all periods of time *T*. With this methodology, all *k* components have a similar weight upon the final value of the Cosine correlation.

**Figure 2:**
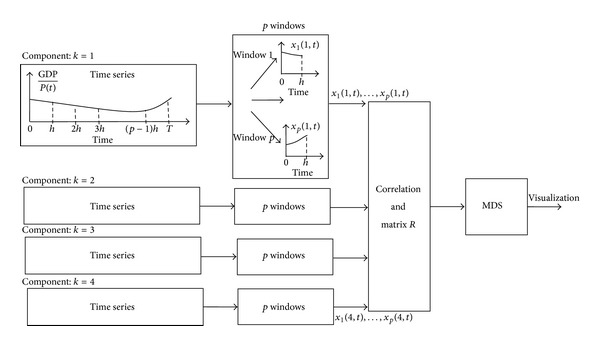
Diagram of the MDS visualization involving *p* windows of *k*_max⁡_ components.

The sampling of the time series with a window *h* converts the *k*-dimensional vector with length *T* into *p* vectors *k*-dimensional with length *h*. In other words, we transform one *T* × *k* dimensional vector into *p* vectors *h* × *k* dimensional. The whole scheme is represented in the diagram of Figure 2.

Equation (1) is the normalized inner product and is called the Cosine coefficient because it measures the angle between two vectors and, thus, often denotes the angular metric [44, 47]. Equations (2) convert to a normalized similarity index the classical Euclidean distance, since max⁡(*δ*_*ij*_^*E*^) consists of the maximum value calculated over the entire set of signals.

We should observe that we are capturing the dynamics of a complex system by means of *k*_max⁡_ economical variables that evolve in time *t*. Each variable has a sampling frequency of 1 year, and, therefore, we have, in fact, discrete signals. Both *r*_*ij*_^*C*^ and *r*_*ij*_^*E*^ capture evolution in discrete-time *t*, but embed the dynamics into a single numerical index. The direct visualizing of time by means of MDS needs the subdivision of the initial data series. A method based on the procrustean transform was proposed in [48]. The Procrustes procedure determines a linear transformation (translation, reflection, orthogonal rotation, and scaling) of the points in a second matrix to best conform them to the points in an initial (reference) matrix. The method proposed in this paper avoids the use of the procrustean transform and guarantees that all time windows are processed simultaneously.

It should be noted that MDS is a mathematical tool that represents, in a low-dimensional map, a set of data points whose similarities are defined in a higher-dimensional space, by means of a symmetric matrix of similarities *R* = [*r*_*ij*_]. In the case of classical MDS (adopted in this paper), the main diagonal of matrix *R* is composed of ones, while the rest of the matrix elements must obey the restriction 0 ≤ *r*_*ij*_ ≤ 1, *i*, *j* = 1,…, *p*. Recall that MDS works with relative measurements, and, consequently, we can rotate or shift the output MDS maps for having a better visualization angle and the conclusions remain the same. The axes have only the meaning, and units (if any) of the measuring index and packages usually apply a heuristic procedure to center the chart. This means that MDS maps are analyzed on the basis of proximity of points and comparison of the resulting cloud of points (e.g., [49] applies it for comparing genomic datasets). A common measure for evaluating how accurately a particular configuration reproduces the initial matrix information is the raw stress, so that the smaller its value, the better the fit between the reproduced and observed matrices. Plotting stress versus the number of dimensions *m* (also called scree plot) usually leads to a monotonic decreasing plot, and we can choose the “best dimension” as a compromise between stress reduction and number of dimensions for the map representation. We can also analyze the goodness-of-fit by means of the Shepard diagram, which for a given number of dimensions depicts the reproduced distances against the input data [50]. Therefore, a narrow scatter around the 45-degree line indicates a good fit of the distances to the dissimilarities, while a large scatter indicates a lack of fit.

In the present case, each element of matrix *R* is obtained either with expression (1), or with (2), yielding a matrix of *p* × *p* similarities. The representation consists of *m*-dimensional plots (*m* = 2,3,…), and the consistency of the map is verified by means of the stress and Shepard charts.

## 3. Data Analysis

**Figure 3:**
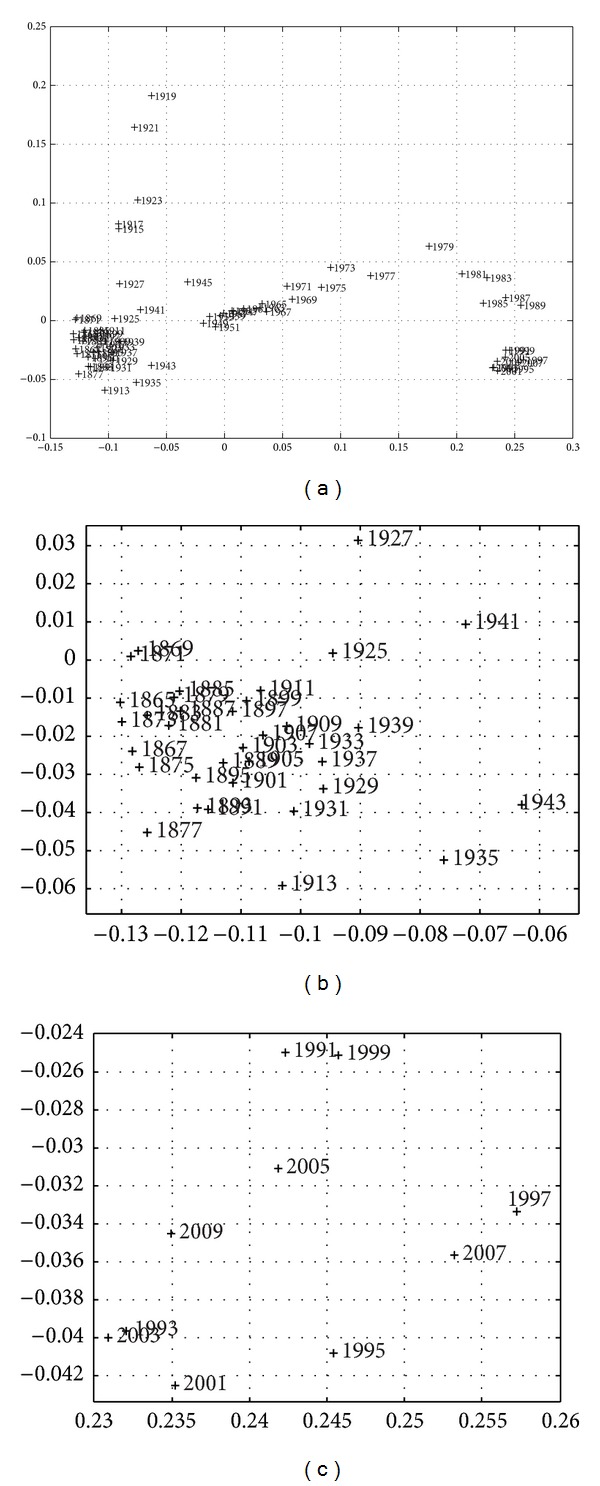
Two-dimensional MDS map of the Portuguese economic evolution, in the perspective of the Cosine correlation *r* ^*C*^, *T* = 1865–2010, and *h* = 2 years. The two charts magnify the two clusters in the left and right lower corners.

**Figure 4:**
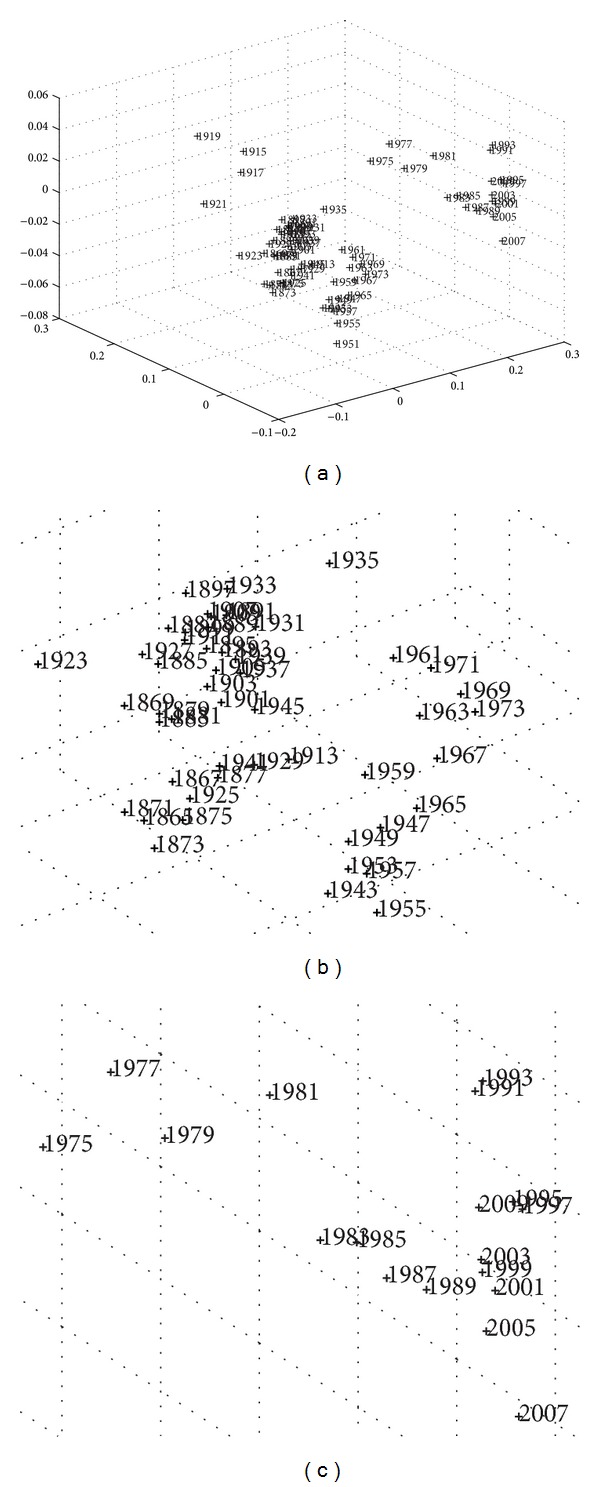
Three-dimensional MDS map of the Portuguese economic evolution, in the perspective of the Cosine correlation *r* ^*C*^, *T* = 1865–2010, and *h* = 2 years. The two charts magnify the two clusters in the middle and right sides.

We start by adopting the Cosine correlation *r*_*c*_ as the measure for similarity. It is considered the data within the historical periods *T* = {1865–2010,1867–2010,1867–2010,1866–2010} for sampling periods of *h* = {2,3, 4,5} years, respectively. Therefore, we obtain symmetric correlation matrices *R* with dimension *p* × *p* = {73 × 73,48 × 48,36 × 36,29 × 29}, respectively. The signals over time are vectors with dimension *k*_max⁡_ = 5, whose composition consists of the normalized values for *k* = {GDP,  Exports,  Imports,  Fiscal  Revenue,  Effective  Public  Expenditure} where “normalized” means the ratio of the absolute value by the total population at that time. Based on this information, MDS provides the *m* = {2,3} dimensional maps represented in Figures 3 and 4, where the symbol + denotes a point and the numerical label indicates the beginning of the corresponding time period. In other words, only the starting year is depicted, so that the label does not significantly disturb the graphic representation.

**Figure 5:**
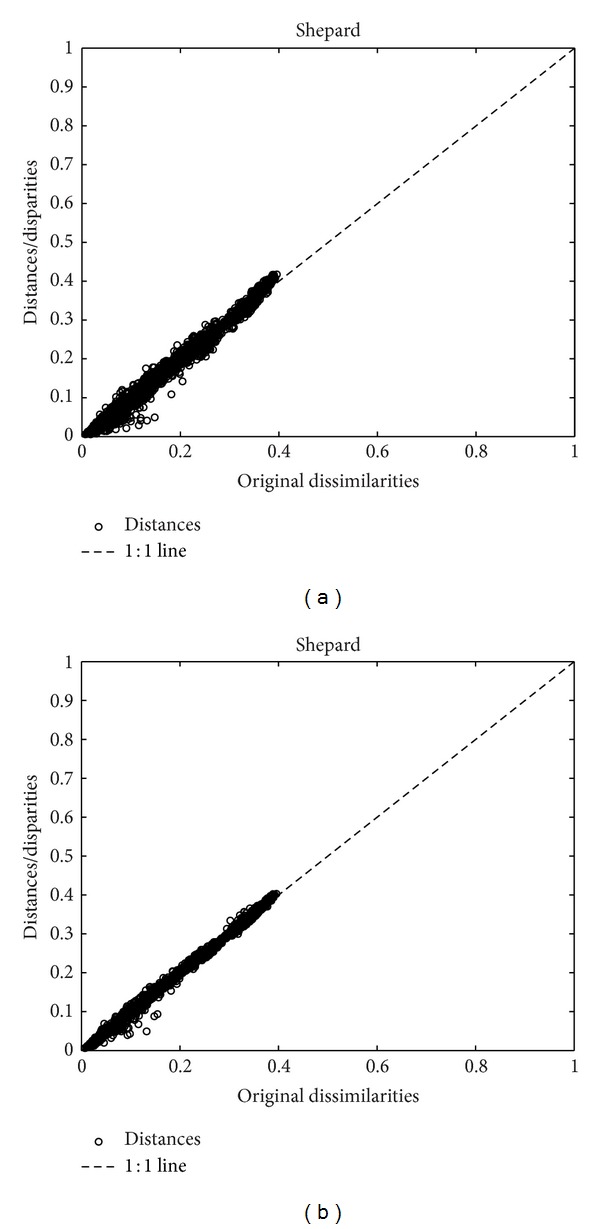
Shepard diagrams of the MDS representation of the Portuguese economic evolution, with *r* ^*C*^, *T* = 1865–2010, and *h* = 2 years for the 2d and 3d maps.

**Figure 6:**
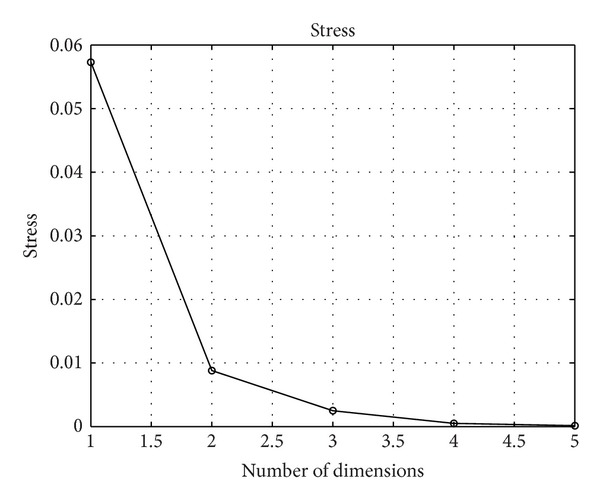
Stress plot of the MDS representation of the Portuguese economic evolution, with *r* ^*C*^, *T* = 1865–2010, *h* = 2 years.

The adjustment quality of the MDS fit with these plots is excellent, as can be seen in the Shepard diagrams (Figure 5) and stress plot (Figure 6). We determine that a two-dimensional space is appropriate for the mapping because the fitting quality is considerable while representations with higher-dimensional *m* add only a marginal improvement.

**Figure 7:**
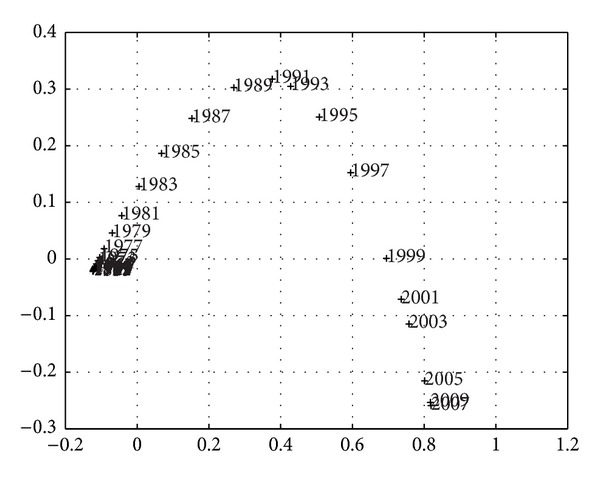
Two-dimensional MDS map of the Portuguese economic evolution, in the perspective of the Euclidean distance *r* ^*E*^, *T* = 1865–2010, *h* = 2 years.

The exercise is repeated using the Euclidean distance *r* ^*E*^ leading to the 3-dimensional map shown in Figure 7. We must observe that at first sight we have a completely different map. Nevertheless, this result is common when varying the measuring index, and, in fact, analyzing MDS maps calls for comparing relative positions and clusters. A detailed visualization of the map confirms the results when using the Cosine correlation. In any event, it is important to reproduce the plot to confirm the robustness of the method in order to draw conclusions.

**Figure 8:**
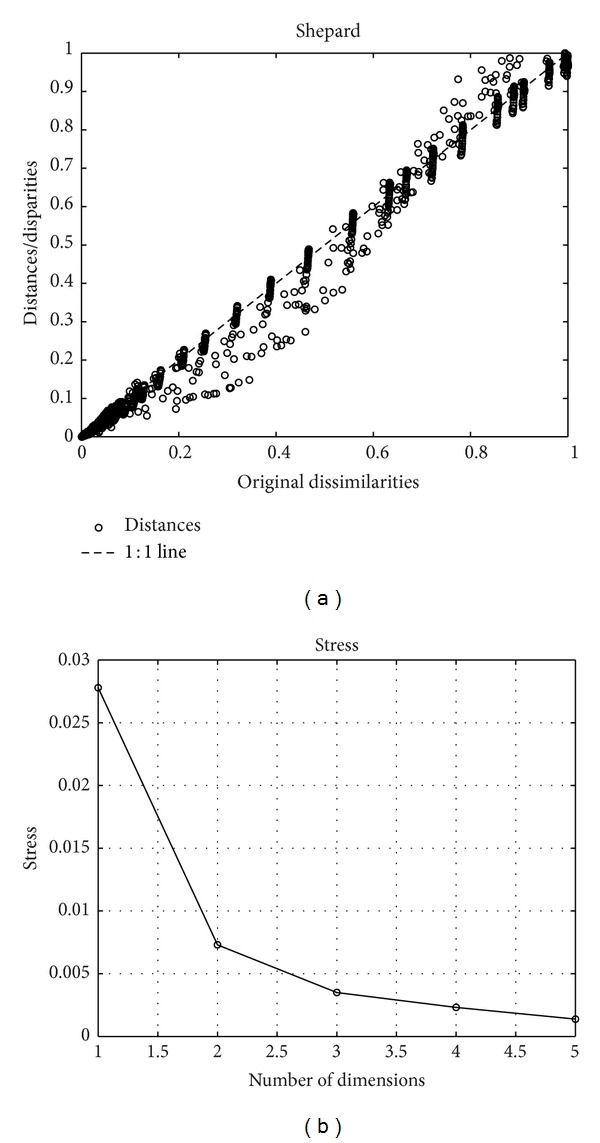
Shepard diagram of the two-dimensional and stress plot of the MDS representation of the Portuguese economic evolution with *r* ^*E*^, *T* = 1865–2010, and *h* = 2 years.

Shepard diagram and stress test (Figure 8) were also investigated, demonstrating that the *m* = 2-dimensional space is an efficient choice in implementing the fit quality. 

**Figure 9:**
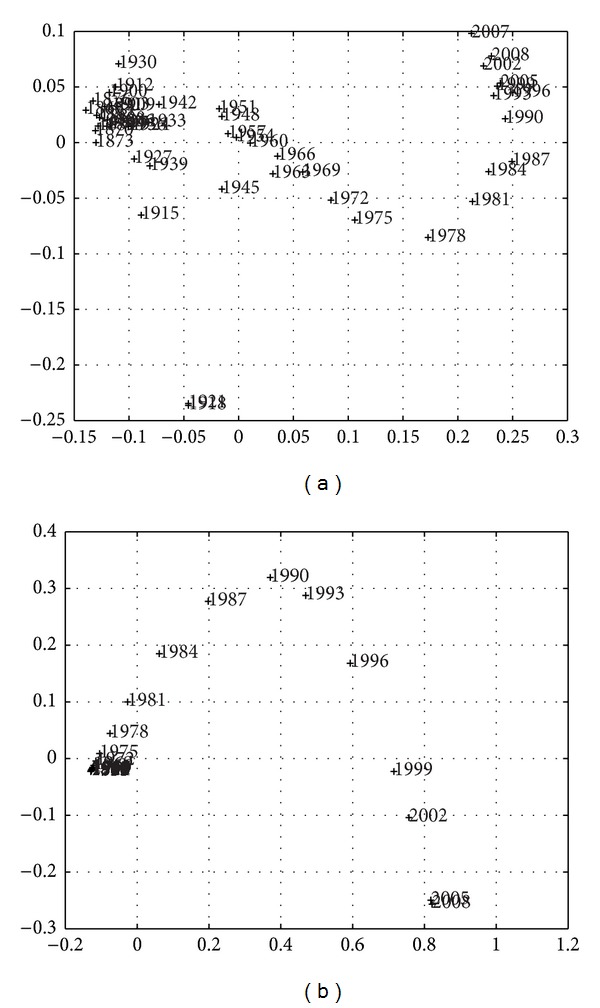
Two-dimensional MDS maps of the Portuguese economic evolution: *T* = 1865–2010, *h* = 3 years for *r* ^*C*^ (a) and *r* ^*E*^ (b).

**Figure 10:**
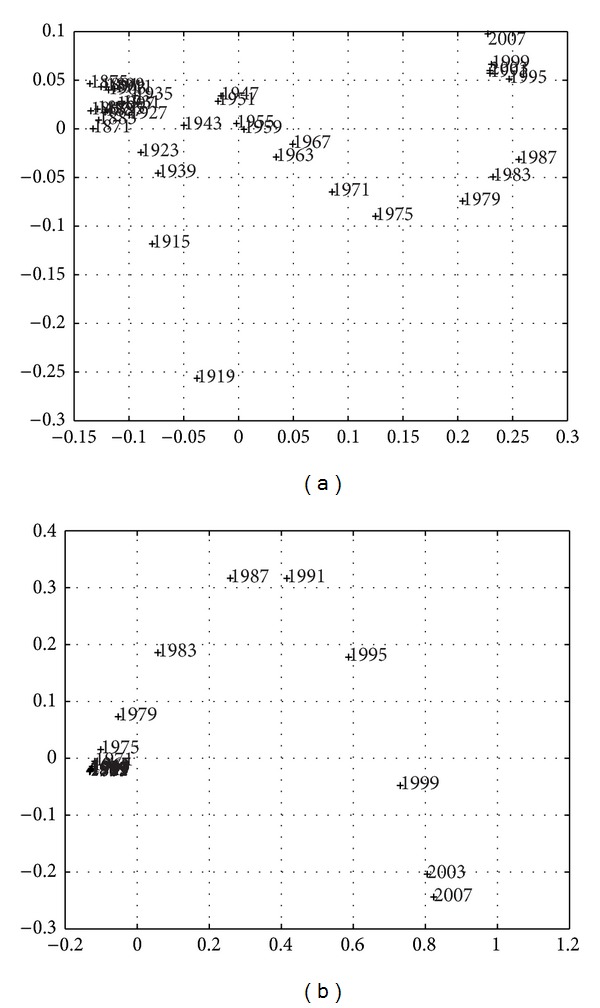
Two-dimensional MDS maps of the Portuguese economic evolution: *T* = 1867–2010, *h* = 4 years for *r* ^*C*^ (a) and *r* ^*E*^ (b).

**Figure 11:**
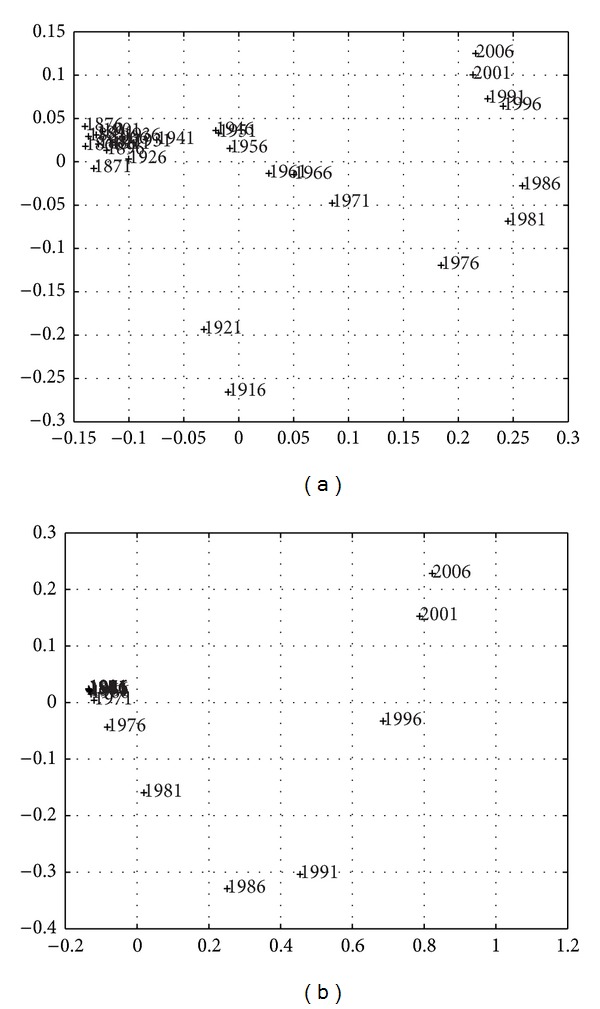
Two-dimensional MDS maps of the Portuguese economic evolution: *T* = 1867–2010, *h* = 5 years for *r* ^*C*^ (a) and *r* ^*E*^ (b).

Larger sampling periods *h*, over approximately the same time length *T*, produce a smaller number of points and simpler MDS maps, but the instantaneous nature is gradually replaced by a smoothing averaging over *h*. Figures 9–11 depict the 2-dimensional MDS maps for *h* = {3,4, 5}, *T* = {1867–2010,1867–2010,1866–2010}, and the indices *r* ^*C*^ and *r* ^*E*^.

Overall, we obtain the same conclusions as previously, but, as expected, we obtain a compromise between resolution (in time) and simplicity (number of points). Another visualization technique is the dendrogram [51], which captures the information content in matrix *R* in a different graphic layout.

The dendrogram is a visual representation of the correlation between data. The individual spots are arranged along the dendrogram and referred to as leaf nodes. Clusters are formed by joining individual leafs, or leaf clusters, with the join point referred to as a node. The horizontal axis is labelled distance and refers to a distance measure between leafs or leaf clusters. The distance *d* measure between two clusters is calculated as follows: *d* = 1 − *r*, where *r* denotes the correlation between leaf clusters.

**Figure 12:**
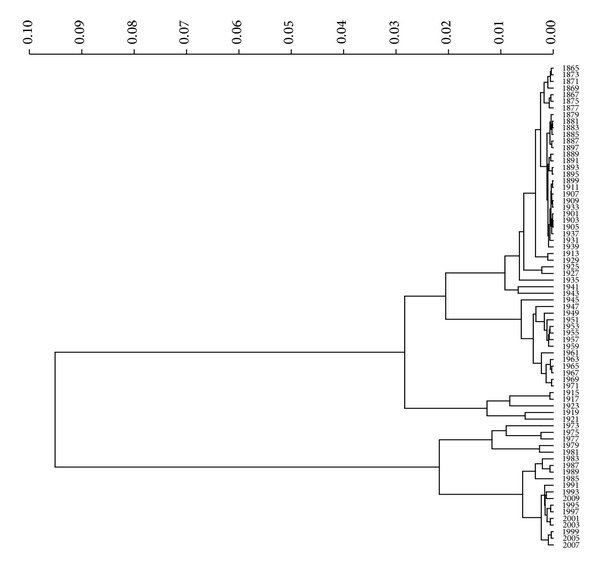
Dendrogram of the Portuguese economic evolution, in the perspective of the Cosine correlation *r* ^*C*^, *T* = 1865–2010, and *h* = 2 years, clustering algorithm: unweighted average.

Figure 12 depicts the dendrogram for the case of *r* ^*C*^, *T* = 1865–2010 and *h* = 2 years. It is straightforward to compare Figures 3, 7, and 12 to conclude that while having the same results, MDS is a good technique for visualizing the information.

**Figure 13:**
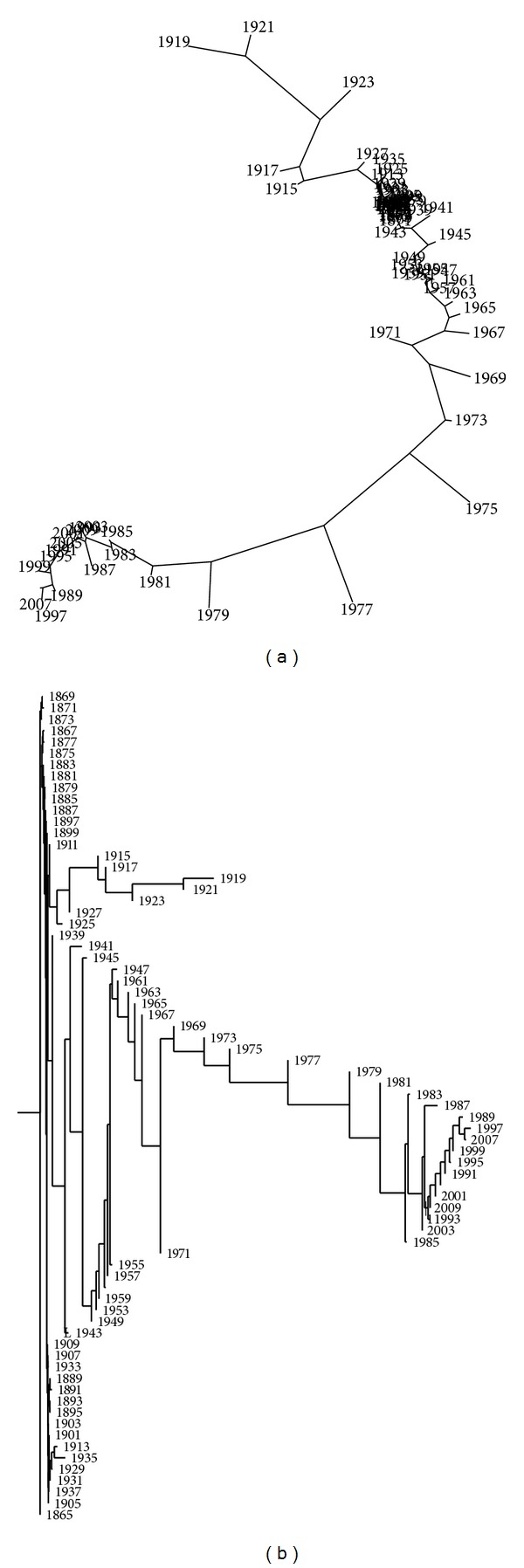
Trees of the Portuguese economic evolution, in the perspective of the Cosine correlation *r* ^*C*^, *T* = 1865–2010, *h* = 2 years, method “neighbor”, (options “drawtree” and “drawgram”).

**Figure 14:**
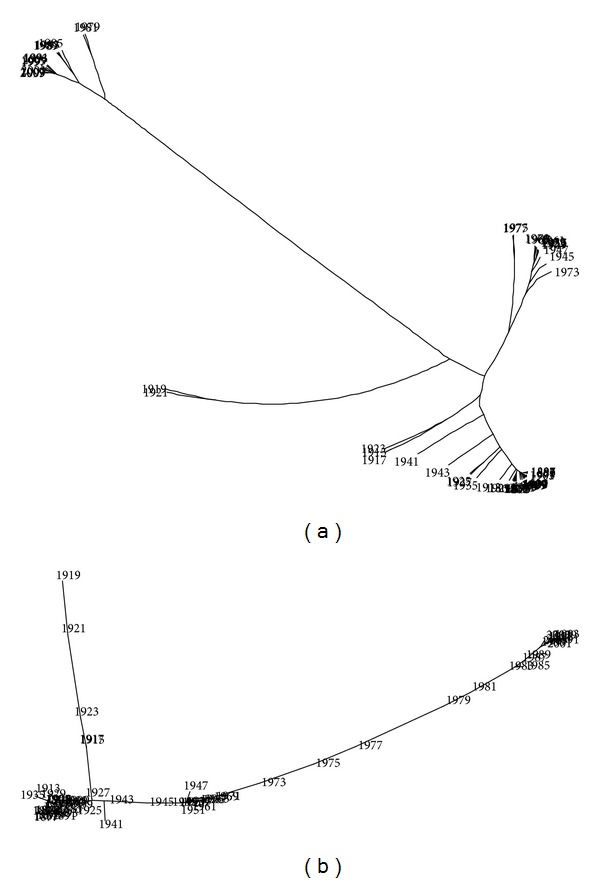
Trees of the Portuguese economic evolution, in the perspective of the Cosine correlation *r* ^*C*^, *T* = 1865–2010, *h* = 2 years, methods “kitsch” and “fitch” (option “drawtree”).

While visual representations are not the main issue addressed in this paper, it is worth mentioning briefly some other visualization techniques. Figures 13 and 14 show the trees generated by package Phylip [52] with method “neighbor” (options “drawtree” and “drawgram”) and methods “kitsch” and “fitch” (option “drawtree”), respectively. These algorithms produce several types of different trees based on the same correlation matrix, trying to fill the two-dimensional space with an efficient visualization technique. In all cases, we get approximately the same conclusions (since the correlation matrix *R* is identical) but with distinct degrees of efficiency.

## 4. Discussion of the MDS Plots

Three large clusters of periods indicate that the period of the First World War and its aftermath was quite dissimilar from any other period in Portugal. This suggestion is quite accurate for 1915–1925, and particularly for 1915–1921. These were the times of large economic and financial difficulties, and their immediate aftermath [53]. High public expenditure for military purposes plagued Portugal since the beginning. Although joining the Allies only in 1917, military operations were required to protect colonial African territories from German attacks. There were low levels of exportation because of the interruption of land and ocean transportation, and low levels of imports for the same reason, which meant market shortage and disruption, as well as large government rotation [18].

Second World War times were less dramatic and relatively similar to the hardships of Great Depression, a conclusion that is quite plausible for similarity. The Great Depression and the Second World War surely hurted the Portuguese economy in different ways, but historians believe that the large weight of agricultural production and self-sufficiency were positive features that mitigated the effects of international trade decline and commercial closure, with the exception of losses from the decrease of colonial raw-material prices [54]. Although the Allied Victory in the war preserved Portuguese rule over the colonial territories, tropical crops were too abundant during the Depression, and prices fell, as did the contribution of reexportation to the Portuguese balance of payments. In spite of crisis synchronization among industrial countries [55], Portugal's small participation in the international markets protected the economy from the effects of depressed exportation prices and from the outcome of Atlantic trade disruption due to submarine attacks. Moreover, Portugal remained neutral throughout the conflict, and could even benefit from tungsten exports to both sides of the conflict (Germany and Allies), according to demand opportunities.

These conclusions are important for understanding that such globally difficult times were not much more severe than the traditional nineteenth-century crises. Looking at Figures 3(a) and 13(b), which show the same MSD points for *h* = 2 years, we may recognize the 1867–1969 crisis, that historians blame on low agricultural production resulting from poor weather conditions, the adverse effects of the Paraguay war on the Portuguese economy, and the knock-on effect of the downturn in the Brazilian economy. Exports to Brazil and emigrants' remittances from Brazil, which usually contributed to the balance of payments, were now very low [56].

Two clusters identify nineteenth-century times (more visible in Figure 3). The smaller one includes happier periods of large public deficits supported by foreign loans in gold-standard times, in which there was easy access to international capital markets to build collective infrastructures, according to the available historical knowledge. This was a public-goods provision policy, dictated by the political blueprint introduced in the 1850s, the so-called Fontismo, evoking the name of the Portuguese politician António Maria Fontes Pereira de Melo. Having assumed the positions of ministers of public works, commerce and industry, finance, navy and overseas and having performed the position of prime minister for periods of office, Fontes assumed a special political role in implementing a rail network to foster Portuguese modernization [57].

According to Figure 3, the most dissimilar period in this cloud is the 1873–1875 euphoria. The largest cluster includes the more difficult crises that are responsible for high government rotation, such as the 1875–1877 panic, the 1887–1892 problems that culminated in abandoning the gold-standard (in 1891), and the 1892 sovereign-debt crisis that led to a partial bankruptcy [58]. Currency depreciation increased the debt burden because debt was expressed in foreign currencies and had to be paid with the national depreciated currency, a genuine *original sin* (as Flandreau and Sussman [59] argue). This partial Portuguese bankruptcy in 1892 consisted of a forced decrease of public debt interest to 1% and a suspension of amortization. It was declared by a government decree on the 13th of June 1892, in the wake of the Baring crisis. The Baring Brothers bank, a traditional lender to the Portuguese government, was suffering from the Argentina and London crisis, which historians attribute to intensified competition among leaders [58]. Short-run loans from abroad, usually received as floating debt, were no longer available because of the South American crisis. This was coupled with a currency “mismatch,” a *twin crisis,* which explains why the payment of interest and amortization could not be achieved. Mitchener and Weidenmier [60] document 46 debt defaults by 25 different countries out of roughly 40 to 50 sovereign countries between 1870 and 1913, while Suter [61] counts 72 default episodes between 1820 and 1913, indicating that situations of this kind were widespread, particularly among capital-poor countries throughout the nineteenth century. Soaring public expenditures and public debt in the nineteenth century are the other face of the rising globalization.

Looking for conclusions for the twentieth century, the large cluster of the 1950s to 1973 in Figure 3 describes the most successful period of modern economic growth in Portugal, which was based on budget equilibrium and large exports to the colonial empire in the 1950s, or on small (and disguised) public deficits to support the colonial war, and large exportation to EFTA in the 1960s [18]. According to Smith [62], the establishment of people in the third colonial empire (in Africa), coupled with industrialization, allowed Salazar to balance the budget throughout the four decades of his government and to pay down the public debt that had accumulated from the *Fontismo* days to the moment before he came to power, and it accounts for the first phase of significant economic growth in Portugal.

Quite apart is the revolutionary period of 1975–1977 in Figures 4(a), 4(c), and 13(a). This period is related to the difficult context of the first oil shock,  the 25th April military revolution of Carnations, and decolonization. An increased population (thanks to the half-a-million return flow of people from the empire) and implementation of democracy led to the need for support in the form of the first International Monetary Fund (IMF) loan. According to Figure 3(a), the 1981–1983 crisis is also quite special, and the need for the second IMF support is usually related to the second oil shock and to political hesitations in designing an economic blueprint for Portugal, after a large communist influence on governance and collective life following the Carnation revolution. Portugal joined Europe and prosperity returned to the Portuguese economy, thanks to economic integration and large budget deficits in a context of easy access to international financial markets for government borrowing.

The special character of the current crisis is clearly visible and identified, as both periods are located quite close and rather distant from any other periods, both in the MDS maps (Figures 3, 7, 9, 10, and 11), reflecting the high values of budget deficits and government debt, and in consequence of the low revenues from exports and high cost of imports. Repeating Eichengreen et al. [3], countries may suffer from the *original sin* of accumulating foreign public debt in globalization, making it very difficult to manage the debt service; an argument that Bordo [4] finds for 30 countries, including Portugal in the period 1880–1914, to conclude on the dramatic character of twin crises (debt and currency crises). This is, again, the special character of the current Portuguese crisis.

**Figure 15:**
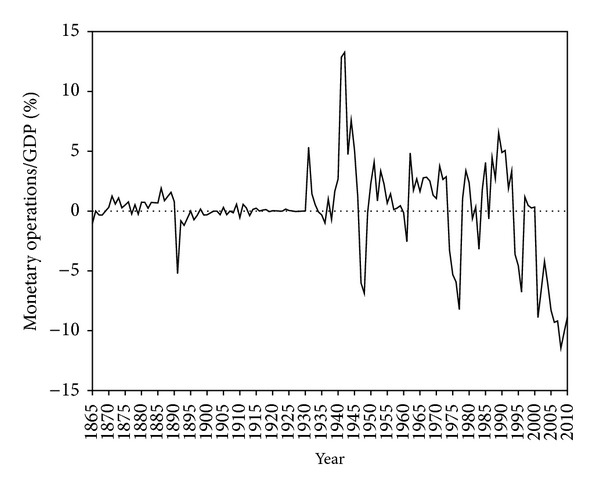
The balance of payments performance: monetary operations/GDP (sources: before 1998 [25]. For the last years [26, 27]).

Looking at the crises identified we may distinguish those that are more related to balance-of-payments problems from those that are more related to government budget deficits. Balance of payments deficits (as a percentage of GDP) were never as dramatic as they are today. As Figure 15 shows (with deficits in the negative vertical axis), discipline in the balance of payments was the rule from 1865 to the 1990s, with few exceptions.

The first significant imbalance occurred in the nineteenth-century gold-standard: 5% of GDP in 1891-1892 was enough to require the suspension of convertibility and the exclusion of credit from international capital markets in 1891, followed by the partial bankruptcy of 1892. The second occurred in 1946-1947, which obliged the Portuguese government to accept the Marshall Plan offer, even after Salazar's declared opinion of rejection. The third occurred in 1961, the year the colonial wars began. The last two before the current new-millennium global crisis occurred at the beginning of the democratic regime because of the two oil shocks, requiring two IMF loans. The current situation is the most dramatic in the entire 150-year analysis, as not only did it come in the mid-1990s (and after a recovery it persists throughout the new millennium), but also dipped below 10% of GDP.

**Figure 16:**
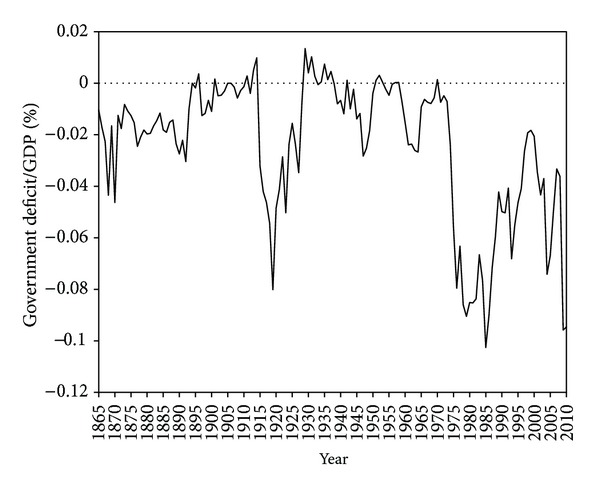
The government budget performance (surpluses on the positive axis and deficits on the negative axis): government deficit/GDP (%) (sources: before 1998 [25]. For the last years [26, 27]).

To make the picture even darker, the central-state government budget mismatch has no parallel in the past, and the First World War was a mild-problem period in comparison with the democracy disarray of 1975–2010, as Figure 16 reveals. As the picture only considers the central state deficit, it is fair to recognize that the real government deficit is not so dramatic because social security and government entrepreneurship sectors are missing here. However, this is the most reliable long-run indicator to preserve homogeneous historical comparisons. 

 Political, literary, and philosophical discussions on decadency come to the fore of the Portuguese cultural scene whenever severe financial problems afflict the economy. More than reflecting on such a theme, it is useful to consider that four major periods may be identified throughout the last 150 years, according to the dendrogram and trees and strong Cosine correlation (while Figure 1 timeline considers the main political and financial features).

The 1865–1891 period illustrates how accumulated foreign borrowing led to the end of the gold-standard in 1891, government bankruptcy in 1892, and slow economic growth. These events were a result of irresponsible borrowing to finance the public works that were considered vital for modernization and industrialization, as it was impossible to impose a level of taxation on the population to support such projects. When financial tensions are too harmful for economic growth and social peace, institutional changes may occur, not only accelerating government turnover (making for short and weak governance periods), but also changing the general political and constitutional framework of the country.

The 1892–1925 period: the Portuguese bankruptcy may be considered as an attempt to decrease the weight of public-debt service in the budget, through a decrease of the interest rate in order to reduce it to values close to the rate of GDP growth. Difficult negotiations with lenders for a conversion of the foreign debt lasted until 1902. In spite of a better economic growth, this financial disaster was laid at the feet of the political regime and especially the monarchy, leading to the victorious Republican Revolution that cast off the royal family and the monarchist regime in 1910. More budget deficits throughout the First World War and the 1920s difficulties exacerbated crises, exhausted the Republican political legitimacy, and led to a new political regime that was born in the May 1926 military putsch.

The 1926–1974 *Estado Novo* financial orthodoxy, with long-term payment of accumulated public debt, required budget control and fiscal system adjustments. They legitimized this political regime and produced the most impressive economic growth path in Portugal's history, both under Salazar and Caetano's governance phases [32].

Portuguese democracy, from the 1974 Carnation revolution until today, illustrates the weakness of fiscal politics [63]. Theories on social consent also ask for a serious scientific character for fiscal systems and an image of political honesty (nineteenth-century England, Peel and Gladstone's efforts to present such an image of the British fiscal system to taxpayers are very well known [64]). In spite of optimistic views on the future of Europe [65], there is a point that political elites may use to convince citizens to accept, or at least tolerate, higher tax burdens [66]. Otherwise, sovereignty alienation or abandoning the Euro may come about, and are high-probability events, at this time.

Some of these considerations may involve ethical, social, and political developments, and, as a result, researchers can discuss their value due to subjectivity. However, the applied methodology, by adopting quantitative analysis indices and a robust visualization technique, leads to compelling conclusions, allowing a substantive assessment of the different periods of economic crisis.

## 5. Conclusions

This paper adopted the MDS analysis for analyzing similarity in business-cycle crises in the Portuguese economy over the last 150 years. The method proved to be highly efficient and accurate in plotting different clusters of crises and in separating out the current Portuguese difficulties. The final result is a coherent overall picture of the phenomena under consideration.

The current Portuguese crisis will draw out how it will be possible for the country to manage the challenge of maintaining its monetary credibility and, hence, its access to foreign capital [67]. It will also make clear how difficult it is to earn the seal of approval from the powerful central banks of the global economy because of violations of “the rules of the game” resulting from persistent government budget deficits.
